# The Influence of Lycopene, [6]-Gingerol, and Silymarin on the Apoptosis on U-118MG Glioblastoma Cells In Vitro Model

**DOI:** 10.3390/nu12010096

**Published:** 2019-12-30

**Authors:** Justyna Czarnik-Kwaśniak, Konrad Kwaśniak, Paulina Kwasek, Elżbieta Świerzowska, Agata Strojewska, Jacek Tabarkiewicz

**Affiliations:** 1 Centre for Innovative Research in Medical and Natural Sciences, Medical Faculty of University of Rzeszow, 1a Warzywna St., 35-310 Rzeszow, Poland; jacek.tabarkiewicz@gmail.com; 2 Department of Human Immunology, Medical Faculty of University of Rzeszow, 1a Warzywna St., 35-310 Rzeszow, Poland; konkwasniak@gmail.com (K.K.); paukwasniak@gmail.com (P.K.); ela.swierzowska@gmail.com (E.Ś.); agata.strojewska1994@gmail.com (A.S.)

**Keywords:** glioblastoma multiforme, Annexin V, mitochondrial membrane potential changes, caspase-3/7, apoptosis

## Abstract

Background: Lycopene, gingerol, and silymarin have a potential anticancer activity in many types of neoplasms. Healthy lifestyle and proper diet are associated with a reduced risk of cancer and other diseases. Increasingly, clinical research focuses on the mechanisms of action of natural compounds and their impact on the development of cancer. The aim of the present study was to determine the effect of lycopene, gingerol, and silymarin on apoptosis, mitochondrial potential and caspase-3/7 activity in the U118-MG cell line. Methods: Human glioblastoma cells were incubated with lycopene, [6]-gingerol, and silymarin for 24 and 48 h. Apoptosis was monitored using the Annexin V labelling, caspase-3/7 activity, and early hallmark of apoptosis were determined with mitochondrial membrane potential changes. Results: Our data showed a significant decrease in the viability glioblastoma cells U118-MG after 48 h treatment with lycopene, [6]-gingerol, and silymarin. Conclusions: Our data could confirm the stimulative effects of used compounds on apoptosis and changes in mitochondrial potential in a dose-dependent manner.

## 1. Introduction

Primary brain tumors account for about 2% of all tumors, and the most common are gliomas. Depending on the age of patients, 40–90% turn out to be malignant. Glioblastoma multiforme (GBM) is the most common primary malignant brain tumor, and it is associated with poor prognosis. Despite advances in treatment modalities, it still remains incurable [1,2,3]. Therapies directed against glioma growth factors and their receptors or related to cell metabolisms are currently at the stage of clinical trials. The angiogenesis inhibitors are considered to be promising medication in GBM [2]. Moreover, many research groups are focused on therapeutic agents, which could unblock the mechanisms leading to cancer cells’ self-destruction and to stop their uncontrolled proliferation. The most effective method of tumor cell elimination is induction of apoptosis [4,5,6]. Lifestyle has a major impact on the development of most tumors. About 60% of new cancer cases are caused by smoking cigarettes and improper diet, and it is estimated that about 30–40% of tumors can be prevented only by diet [7]. Protective elements in the antineoplasm diet include selenium, folic acid, vitamin D, antioxidants, lycopene, gingerol, and silymarin [4]. In cancer prevention, great emphasis is placed on the consumption of vegetables and fruits. They contain fiber and antioxidants of particular importance not only in the prevention of cancer, but also in the prevention of cardiovascular disease and type II diabetes [7]. The combination of oncological therapy and dietotherapy may have an impact on the well-being and comfort of patients. In the oncological treatment process, dietotherapy is based on the assessment of the patient’s nutritional status, cancer location, and progress [5]. Therefore, effective methods for the prevention and treatment of glioblastoma multiforme should be developed. Tomatoes and their products are an important source of antioxidants, such as phenolic compounds, as well as carotenoids, including lycopene. Important components of tomato are vitamins, especially rich in water soluble vitamin C, thiamine, niacin, riboflavin, pantothenic acid, folic acid, and biotin [6]. Lycopene is a polyunsaturated chain fat-soluble compound derived, inter alia, red pigment produced by plants and some microorganisms. It belongs to the class of organic compounds known as carotens (water solubility at 0.0004 g/L). Depending on the variety, tomatoes may contain from 3.1–7.74 mg/100 g of lycopene; however, the largest amount can be found in tomato concentrates 38.0–49.3 mg/100 g [8]. The health-promoting properties of lycopene are mainly related to its strong antioxidant properties and epigenetic regulation of gene expression. This aroused interest in tomato as a food with potential anticancer properties. There is a correlation between the regular consumption of fresh tomatoes and their products, and a reduction in the incidence of various types of cancer [9,10,11]. Medical ginger has a number of applications and properties; the most well-known action is improving symptoms of dyspepsia, such as indigestion, nausea, and even unresponsive vomiting of pregnant women. Ginger also has a tradition in Indian and China medicine; it was used as an anti-inflammatory agent for musculoskeletal diseases [12].

It contains phenolic substances, aromatic ketones known as gingerols, which are thought to be the most pharmacologically active components [13]. Moreover, [6]-Gingerol (1-4′-hydroxy-3′-methyloxyphenyl-5-hydroxy-3-decanone), which belongs to polyphenols (water solubility at 0.08g/L (80 mg/dm^3^)), is one of the major ingredients of ginger, and it represents several mechanisms of action, such as an antipyretic and analgesic effects mediated by inhibition ofprostaglandin and leukotrienes biosynthesis, platelet aggregation. In an in vivo study, ginger oil at a single oral dose of 33 mg/kg supressed severe chronic adjuvant arthritis in rats [14]. Through the induction of apoptosis, gingerol has been shown to exhibit anticancer activities. Due to its strong anti-inflammatory effect, it is considered to be associated with its cancer chemopreventive potential [14]. Silymarin is a polyphenolic flavonoid isolated from milk thistle, composed mainly of flavonolignans: silibinin, isosilybin, dihydrosilybin, silydianin, and silychristin. It is soluble in water at 77.36 mg/L. Similarly to lycopene and gingerol, silymarin are strong antioxidants, so they are able to scavenge both free radicals and reactive oxygen species, which results in enhancement of cellular antioxidant defense machinery [15,16,17].

Some pharmacological study revealed that silymarin is nontoxic even in the highest doses; therefore, it is safe for treating various diseases [18].

In this study, we have evaluated potential of lycopene, [6]-gingerol, and silymarin to induce apoptosis of the human glioblastoma multiforme cells. We hypothesize that natural compounds in glioblastoma cells may be useful for development of new strategies for cancer and other diseases treatment.

## 2. Materials and Methods

### 2.1. Cell Culture and Chemicals

U-118 MG cells (ATCC^®^ HTB-15™) classified as grade IV of glioblastoma were purchased from the American Type Culture Collection (Manassas, VA, USA). The cells were cultured in Dulbecco Minimal Essential Medium (DMEM) (ATCC, USA) supplemented with 10% foetal bovine serum (FBS) (EurX, Gdańsk, Poland), 1% penicillin, and streptomycin solution (Sigma Aldrich, St. Louis, MO, USA) in an incubator with a humidified atmosphere of 95% air and 5% CO_2_ at 37 °C. Culture media was refreshed every 2–3 days and cells were passaged at 70–80% of confluence using 0.25% acutase enzyme solution (Corning, Polgen, Łódź, Poland). Before sub-cultivation, cells were washed with phosphate buffered saline (PBS) (Biowest, France). Cell morphology was observed with the use of inverted microscope (Zeiss, City, Germany). U-118MG cell line were seeded in flat-bottom 24-well culture plates (VWR, Radnor, PA, USA) in five biological repeats at a density of 4 × 10^4^ cells/well. Cells were allowed to attach for 24 h before treatment.

### 2.2. Treatment of Cells

**Figure 1:**
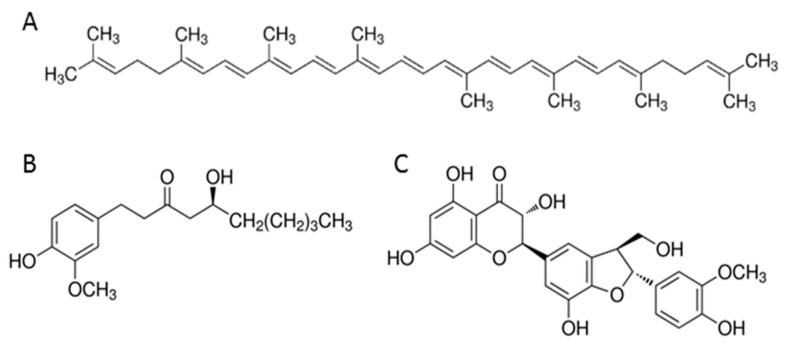
Chemical structure of tested compounds: (**A**) lycopene; (**B**) [6]-gingerol; (**C**) sylimarin.

Lycopene (C_40_H_56_, MW = 536.87 g/mol) at >90% purity, [6]-gingerol (C_17_H_26_O_4_, MW = 294.38 g/mol) at >98% purity and silymarin (C_25_H_22_O_10_, MW = 482.44 g/mol) >95% purity (all of them Sigma Aldrich, St. Louis, MO, USA) (Figure 1) were dissolved in dimethyl sulfoxide (DMSO) (VWR, Radnor, PA, USA) to produce 10 mM lycopene, 100 mM [6]-gingerol, and 100 mM silymarin stock solutions. The microliter volumes of stock solutions were added to the 1 mL culture medium to obtain variable concentrations of studied compounds. The concentration of DMSO was kept 0.5 v/v% by addition of neat DMSO. The control solution was 0.5% DMSO in medium. The apoptotic tests were performed for 0–50 μM lycopene, 0–500 μM [6]-gingerol and 0–200 μM silymarin solutions. The cells were incubated on such media for 24 and 48 h.

### 2.3. Determination of Apoptosis by Annexin V Staining

The apoptosis was measured using the Annexin V Dead Cell Kit (Merck Millipore, Burlington, MA, USA) according to the manufacturer’s protocols. In general, U-118 MG cells grown in 24-well plates were treated with increasing concentrations of lycopene, [6]-gingerol, silymarin, and DMSO for 24 and 48 h. Adherent cells were washed, collected, and incubated with Annexin V and 7-AAD (7-Aminoactinomycin D -dead cell marker) for 20 min in dark at room temperature. The percentages of live, dead, early, and late apoptotic cells were measured with the Muse^®^ Cell Analyzer (Merck Millipore, Burlington, MA, USA).

### 2.4. Detection of the Mitochondrial Membrane Potential

The mitochondrial membrane potential changes were measured with the Muse^®^ Cell Analyzer (Merck Millipore, Burlington, MA, USA) using MitoPotential Assay (Merck Millipore, Burlington, MA, USA) according to the manufacturer’s protocol. Briefly, U-118 MG cells were treated with increasing concentrations of lycopene, [6]-gingerol, silymarin and DMSO for 24 and 48 h. Adherent cells were collected, washed and incubated with the Muse^TM^ Mitopotential Dye, a cationic, lipophilic dye to detect changes in the mitochondrial membrane potential for 20 min at 37 °C in the thermoblock (Eppendorf, Hamburg, Germany). Next, cells were incubated with 7-AAD, an indicator of cell death for 5 min at room temperature.

### 2.5. Quantification of Caspase-3/7 Activity and DNA Electrophoresis

Caspase-3/7 activity was quantified by using the Muse Caspase-3/7 assay kit (Merck Millipore Burlington, MA, USA) according to the manufacturer’s protocol. In short, U-118 MG cells grown in 24-well plates were treated with increasing concentrations of lycopene, [6]-gingerol, silymarin and DMSO for 24 and 48 h. Adherent cells were washed, collected and incubated with the Muse^TM^ Assay Buffer BA, then incubated with Muse^TM^ Caspase-3/7 Reagent, a hallmark of apoptosis for 30 min in the 37 °C incubator with 5% CO_2_ (Panasonic, Gunma, Japan). Afterwards, cells were incubated with Muse^TM^ Caspase 7-AAD, a dead cell marker for 5 min at room temperature. The percentages of live (Caspase-3/7 (−) and 7-AAD (−)), dead (Caspase-3/7 (−) and 7-AAD (+)), apoptotic cells exhibiting Caspase-3/7 activity (Caspase-3/7 (+) and 7-AAD (−)) and late apoptotic/dead (Caspase-3/7 (+) and 7-AAD (+)) cells were assessed using the Muse^®^ Cell Analyzer (Merck Millipore, Burlington, MA, USA). DNA electrophoresis was performed according to protocol described by Herrmann et al. [19].

### 2.6. Statistical Analysis

The experiment was repeated five times and the results of analysis are presented as median. All the statistical analysis including ANOVA (analysis of variance) Friedman and post-hoc tests analysis was performed using Statistica 13.1 PL (Statsoft, Kraków, Poland).

## 3. Results

### 3.1. Effect of Lycopene, [6]-Gingerol, and Silymarin on Apoptosis of U118-MG Glioblastoma Cells Evaluated by an Annexin V Dead Cell Kit

**Figure 2:**
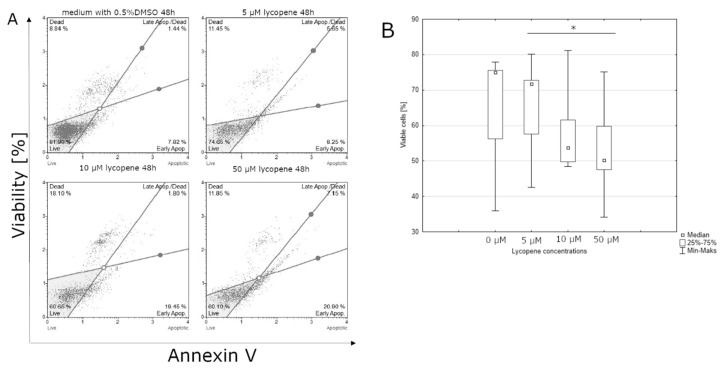
(**A**) lycopene induces apoptosis in the U-118MG cells. Cells were treated with DMSO or various concentrations of lycopene for 48 h. Adherent cells were collected and stained with Annexin V and 7-AAD and events for early apoptotic cells were counted with the Muse Cell Analyzer; (**B**) effect of the indicated concentrations of lycopene on U-118MG apoptosis after 48 h incubation using Annexin V staining. The representative experiment is a median and difference between lycopene concentration and the control evaluated using ANOVA Friedman *p* = 0.02 and post-hoc tests * *p* < 0.05.

**Figure 3:**
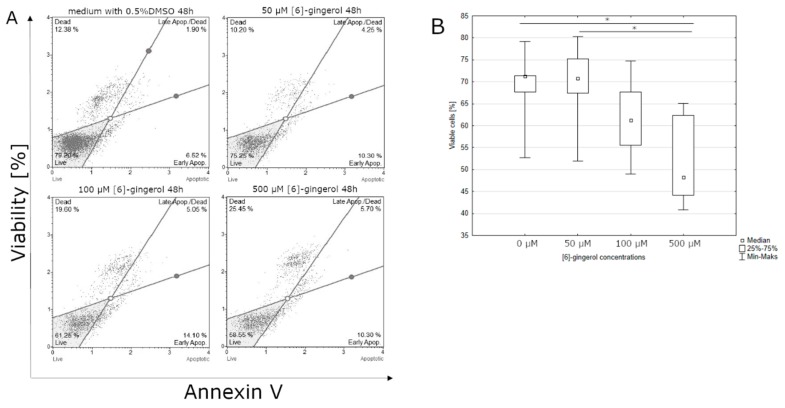
(**A**) [6]-gingerol induce apoptosis in the U-118MG cells. Cells were treated with DMSO or various concentration of [6]-gingerol for 48 h. Adherent cells were collected and stained with Annexin V and 7-AAD and events for live cells were counted with the Muse Cell Analyzer; (**B**) effect of increasing [6]-gingerol concentration on U-118MG living cells percentages after 48 h incubation using Annexin V staining. The representative experiment is a median and a difference between [6]-gingerol concentration and the control evaluated using ANOVA Friedman *p* = 0.029 and post-hoc tests * *p* < 0.05.

**Figure 4:**
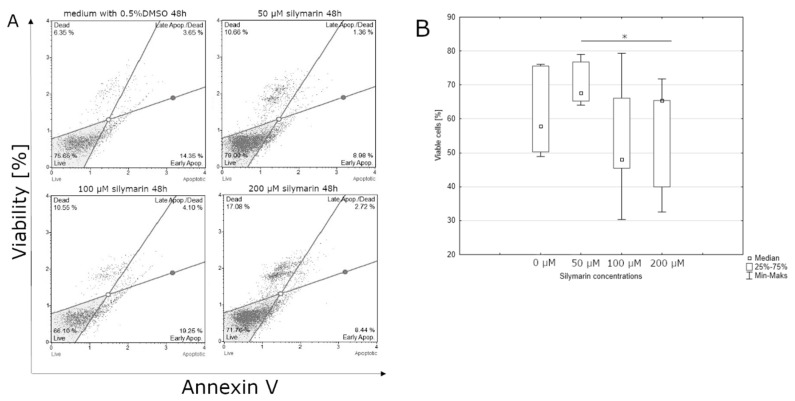
(**A**) silymarin induces apoptosis in the U-118MG cells. Cells were treated with DMSO or various concentration of silymarin for 48 h. Adherent cells were collected and stained with Annexin V and 7-AAD and events for live cells were counted with the Muse Cell Analyzer; (**B**) effect of the indicated concentrations of silymarin on U-118MG living cells after 48 h incubation using Annexin V staining. The representative experiment is a median and a difference between silymarin concentration and the control evaluated using ANOVA Friedman *p* = 0.04 and post-hoc tests * *p* < 0.05.

After 24-hour incubation, there were no significant differences between the percentage of live cells in lycopene treated and non-treated cell culture (data not shown). Lycopene can affect the percentage of cells in the early stages of apoptosis after 48 h incubation. The highest live cell percentages were observed at the control culture. The lowest percentage of live cells was observed at the highest concentration of lycopene (50 μM) (Figure 2). Similar to lycopene, a statistically significant effect on the percentage of living cells was observed after 48 h of incubation with [6]-gingerol, but not after 24 h. The smallest percentage of live cells was observed in the culture incubated with the highest dose of [6]-gingerol (500 μM) while the highest percentage of live cells cultured with the lowest concentration of [6]-gingerol (50 μM) (Figure 3). After 24 h of incubation, the lycopene, [6]-gingerol, and silymarin used in this study were not statistically significant (data not shown). Referring to the above results, silymarin significantly influences the percentage of living cells after 48 h of incubation. The highest percentage of live cells were found in the culture supplemented with 50 μM of silymarin, while the lowest when the dose of 100 μM of silymarin was used. (Figure 4).

### 3.2. Effect of Lycopene, [6]-Gingerol and Silymarin on Apoptosis of U118-MG Glioblastoma Cells Evaluated by a Mitopotential Assay

**Figure 5:**
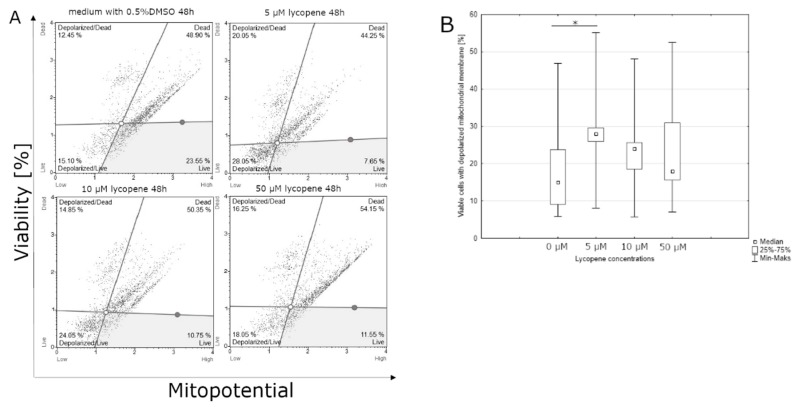
(**A**) lycopene induces the depolarization of mitochondrial membrane after 48 h incubation using mitopotential assay. Adherent cells were collected and stained with 7-AAD and then events for depolarized live cells were counted with the Muse Cell Analyzer; (**B**) assessment of the percentage of viable cells with depolarized mitochondrial membrane after 48 h incubation depending on the concentration of lycopene. The representative experiment is a median and a difference between lycopene concentration and the control evaluated using ANOVA Friedman *p* = 0.01 and post-hoc tests * *p* < 0.05.

**Figure 6:**
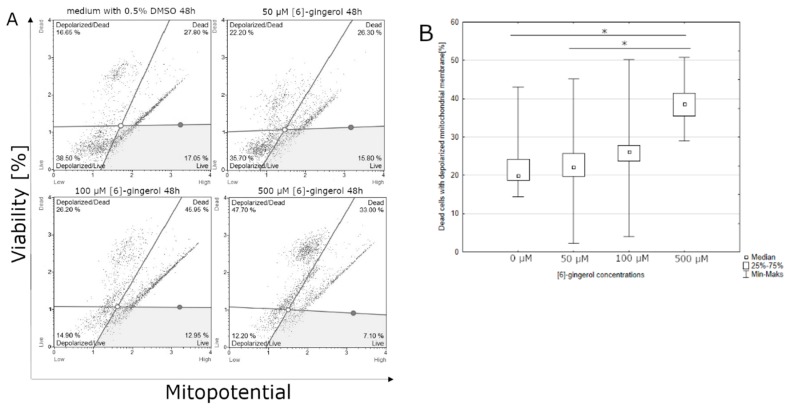
(**A**) [6]-gingerol induces the depolarization of mitochondrial membrane after 48 h incubation using a mitopotential assay. Adherent cells were collected and stained with 7-AAD and then events for depolarized dead cells were counted with the Muse Cell Analyzer; (**B**) assessment of the percentage of dead cells with a depolarized mitochondrial membrane after 48 h incubation depending on the concentration of [6]-gingerol. The representative experiment is a median and a difference between [6]-gingerol concentration and the control evaluated using ANOVA Friedman *p* = 0.013 and post-hoc tests * *p* < 0.05.

Lycopene can affect the percentage of viable cells with the mitochondrial membrane potential depolarization after 48 h of incubation. The highest percentage of viable cells was observed at the lowest dose of lycopene (5 μM); on the contrary, the lowest percentage of viable cells was observed at the highest concentration of lycopene (50 μM) (Figure 5). Significant differences were observed after 48 h incubation with [6]-gingerol on percentage of dead cells with the depolarized mitochondrial membrane of U-118MG cells. The highest percentage of dead cells with depolarized mitochondrial membrane was observed with the highest dose of [6]-gingerol (500 μM), while the lowest percentage of dead cells with depolarized mitochondrial membrane in the control sample (Figure 6). The highest percentage of dead cells with the depolarized mitochondrial membrane has been examined with the intermediate dose of silymarin (100 μM), while the lowest percentage of dead cells with the depolarized mitochondrial membrane was observed at the lowest concentration of silymarin (50 μM). The use of a silymarin doses did not significantly affect the total percentage of cells with the depolarized mitochondrial membrane after 48 h of incubation (data not shown).

### 3.3. Effect of Lycopene, [6]-Gingerol and Silymarin on Caspase-3/7 Activity of U118-MG Glioblastoma Cells Evaluated by Caspase-3/7 Assay

**Figure 7:**
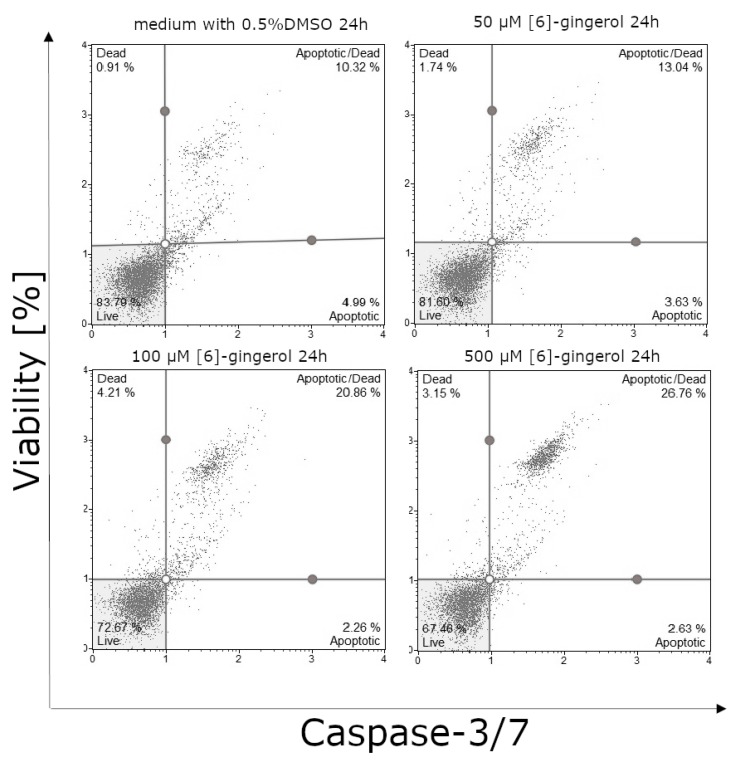
[6]-gingerol induce apoptosis in the U-118MG cells. Cells were treated with DMSO or various concentrations of [6]-gingerol for 24 h. Adherent cells were collected and stained with a Muse^TM^ Caspase-3/7 Reagent and 7-AAD and events for live cells were counted with the Muse Cell Analyzer.

**Figure 8:**
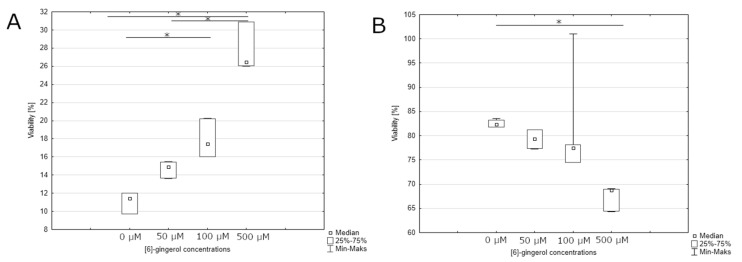
(**A**) assessment of the percentage of late stage of apoptotic or dead cells after 24 h incubation depending on the concentration of [6]-gingerol. The representative experiment is a median and a difference between [6]-gingerol concentration and the control evaluated using ANOVA Friedman *p* = 0.00044 and post-hoc tests * *p* < 0.05; (**B**) effect of increasing [6]-gingerol concentration on U-118MG living cells percentages after 24 h incubation using Muse^TM^ Caspase-3/7 Reagent and 7-AAD staining. The representative experiment is a median and a difference between [6]-gingerol concentration and the control evaluated using ANOVA Friedman *p* = 0.00094 and post-hoc tests * *p* < 0.05.

**Figure 9:**
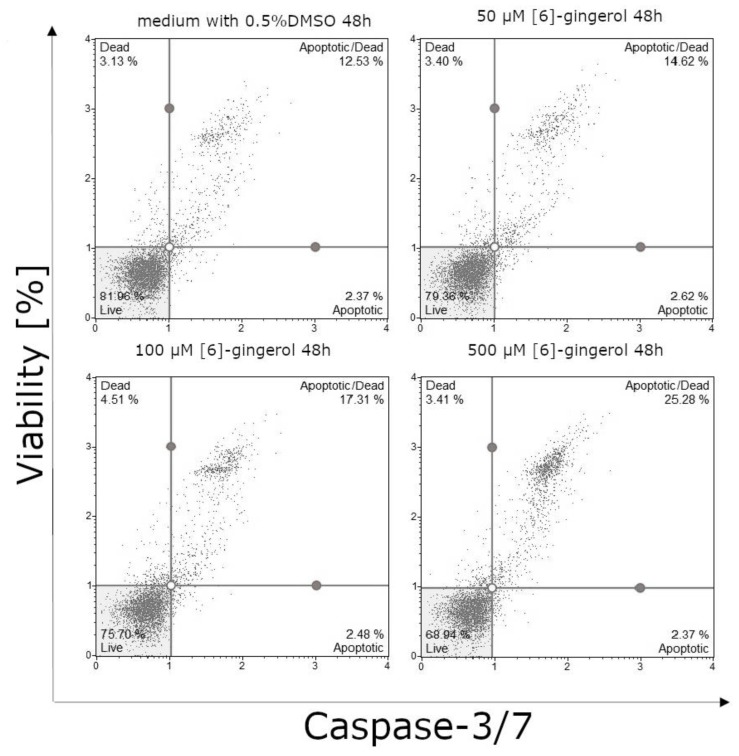
[6]-gingerol induce apoptosis in the U-118MG cells. Cells were treated with DMSO or various concentration of [6]-gingerol for 48 h. Adherent cells were collected and stained with Muse^TM^ Caspase-3/7 Reagent and 7-AAD and events for live cells were counted with the Muse Cell Analyzer.

**Figure 10:**
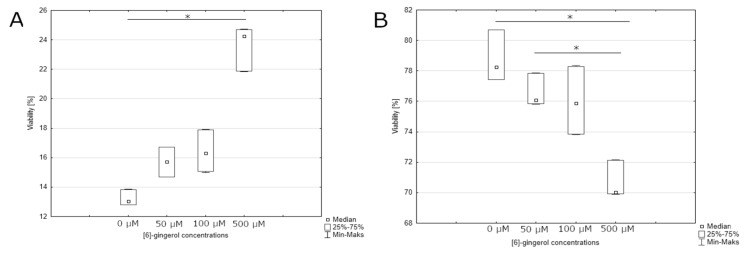
(**A**) assessment of the percentage of late stage of apoptotic or dead cells after 48 h incubation depending on the concentration of [6]-gingerol. The representative experiment is a median and a difference between [6]-gingerol concentration and the control evaluated using ANOVA Friedman *p* = 0.00094 and post-hoc tests * *p* < 0.05; (**B**) effect of increasing [6]-gingerol concentration on U-118MG living cells percentages after 48 h incubation using Muse^TM^ Caspase-3/7 Reagent and 7-AAD staining. The representative experiment is a median and a difference between [6]-gingerol concentration and the control evaluated using ANOVA Friedman *p* = 0.0029 and post-hoc tests * *p* < 0.05.

After 24-h incubation, we observed a statistically significant effect on the percentage of live cells and apoptotic cells in the late stage of apoptosis or dead cells treated by [6]-gingerol. The smallest percentage of live cells was observed in the culture incubated with the highest dose of [6]-gingerol (500 μM) with the highest percentage of live cells at the control culture (Figure 7 and Figure 8A). We noticed similar results on the percentages of apoptotic or dead cells: the smallest percentages of these cells were in control culture, whilst the highest percentages of late stages of apoptotic or dead cells were after incubation with 500 μM [6]-gingerol (Figure 8B). Confirmation of our results are the data presented after 48-hour incubation. The highest percentages of apoptotic or dead cells were observed in culture after stimulation with 500 μM, such as decreasing the viability of U-118MG cells (Figure 9 and Figure 10A,B). After 24-h and 48-h incubation, there were no significant differences between percentage of live cells, late stages of apoptotic or dead cells in lycopene, and silymarin treated and non-treated cell culture (data not shown). Additionally, we confirmed apoptosis by DNA electrophoresis showing typical fragmentation of DNA (Supplementary Figure S1).

## 4. Discussion

In this study, we examined the inhibitory effect of lycopene, [6]-gingerol, and silymarin on the cell viability and stimulatory effect on the apoptosis of U-118MG glioblastoma cells. It is well known that a characteristic feature of the apoptosis process are changes occurring in the structure of the cell membrane. It leads to distortion of the asymmetry in the distribution of membrane phospholipids. In a normal cell, on the membrane surface, neutral phospholipids predominate, including sphingomyelin and phosphatidylcholine. The inner layer is dominated by anionic phospholipids such as phosphatidylserine. In the apoptotic cells, phosphatidylserine is exposed in the outer layer of the cell membrane. This phenomenon is used to label apoptotic cells via Annexin V, which was the ability to preferentially bind to negatively charged phospholipids such as phosphatidylserine [20].

Two intertwining molecular events determine the point from which the irreversible process of cell death begins. These include mitochondrial membrane permeability and caspase activation [21], regardless of the apoptosis pathway [10]. In the case of increase mitochondrial membrane permeability caused by e.g., overproduction of ROS (Reactive Oxygen Spieces) or overloading of organelles with Ca^2+^ ions due to their increased uptake from the cytoplasm, it comes to the creation of megachannels in the mitochondrial membrane and lead to high permeability. The consequence of increasing mitochondrial membrane permeability is the release into cytoplasm of mitochondrial proapoptotic proteins (e.g., cytochrome c, SMAc/DIABLO, and AIF). Cytochrom c released from the membrane interstitial space of mitochondria interacts with the APAF-1 protein and changing its conformation leads to the formation of apoptosome complex that activates caspase-9, which in turn activates executive caspases-3, -6, -7 [11]. Membrane permeability to cytochrome c is considered a critical step in the progression of irreversible apoptosis process, and we can conclude that this is an early stage of apoptosis [22]. Here, we report that lycopene significantly affected the number of viable cells after 48 h incubation, and the percentage of living cells with depolarized mitochondrial membrane was considerably lower at 50 μM than in the control. This may suggest that the cells enter into early stage of apoptosis associated with a change in the structure of the mitochondrial membrane. The total percentage of apoptotic cells markedly increased in the 48-hour incubation with the 50 μM lycopene dose compared to 24-hour incubation. In our study, we did not show statistically significant differences after 24 h incubation with examined substances. Taking into consideration caspase/3/7 activity, we also do not observe statistically significant differences after 24 and 48-h lycopene incubation. Nevertheless, Palozza et al. found that a 24-hour treatment with lycopene resulted in a dose-dependent increase in 7-amido-4-methylcoumarin fluorescence, leading to activation of caspase-3 in prostatic carcinoma LNCaP cells [23]. Puri et al. in their study conducted a randomized placebo trial, in which 50 patients with high-grade gliomas were treated surgically and then treated with oral lycopene 8 mg/day or placebo. Time to progression was longer in the lycopene-supplemented group but not statistically significant [24]. Therapeutic benefits of using lycopene are well known in the case of various cancers. In the study conducted in 1995, authors demonstrated that the consumption of carotenoid was not associated with reduced risk of prostate cancer, but high lycopene intake was related to statistically significant risk reduction. Giovannuci et al. identified lycopene as a carotenoids with the greatest ability to inhibit the development of prostate cancer [25]. In a similar study lasting from 1986 to 1998, 47,367 men were examined and completed a dietary questionnaire every four years. Frequent consumption of tomatoes was associated with a reduced risk of prostate cancer and the consumption of tomato sauce was associated with even lower risk of this type of cancer [16]. Rafi et al. showed that lycopene in combination with a chemotherapeutic agents induced apoptosis in prostate cancer cells [17]. In case of HT-29 colon carcinoma cells, it was shown that lycopene at concentration of 2 μM together with 25 μM eicosapentaenoic acid (EPA) synergistically inhibit tumor cell proliferation. Lycopene and EPA also blocker of Akt/mTOR activation contributing to decreased cell proliferation [26]. In turn, in another study, the effects of lycopene on the proliferation of several human cell lines, including cancer, non-cancer cells were analyzed. The hepatic adenocarcinoma cells showed a decrease in proliferation rate in high doses of lycopene after 24 h and the non-neoplastic pulmonary cells showed a proliferation decreasing at the highest dose 10 mM after 72 hours, compared to the control. Cells of skin cancer, prostate cancer, breast cancer, lung cancer, and noncancerous skin did not show reduced proliferation [27]. In the present study, there were no essential differences in the lycopene glioblastoma proliferation (data not shown), which is in accordance with a study performed by Burgess et al. [27]. Another confirmation of the inhibitory effects of lycopene is the data presented by Karas et al., in which stimulation of IGF-1 (insulin-like growth factor-1) breast cancer cell growth has been significantly reduced by lycopene at 3 μM concentration. It was concluded that the inhibitory effect of lycopene on MCF7 cell growth is not due to carotenoid toxicity, but rather to interference in IGF-1 receptor signalling and cell cycle progression [28]. Lycopene exerted a significant dose-related effect on the proliferation capacity of the myeloid leukemia, colon cancer, and Burkitt’s lymphoma cell line. This effect was observed in lymphocytic leukemia cells only at the highest dose (4 μM) used in that study. Increased apoptosis was found after incubation of colon cancer cells with 2 μM/mL and 4 μM/mL of lycopene as well as in Burkitt’s lymphoma cells after incubation with 2 μM. It has been concluded that the antiproliferative activity of lycopene on tumor cells and its effect on the rate of apoptosis depends on its dose and type of malignant cells [29].

Gingerol, besides lycopene, exhibited potential anticancer activity in many types of cancer. Our results suggest that gingerol also has a significant influence on the depolarization of the mitochondrial membrane of tumor cells. At the highest concentration (500 μM), the percentage of dead cells after 24 h was 70%, and, after 48 h, it was 60%. The percentage of proliferating cells was lowest in culture with 50 μM [6]-gingerol; however, the values were comparable (data not shown). Similar results were obtained after a 48 h incubation. We presented, after 24-hour incubation, the highest percentages of late stage of apoptotic cells with caspase-3/7 activity or dead cells at the highest concentration of [6]-gingerol. We can suggest late apoptosis events even at the lowest concentration. Mostly, the dose-dependent activation of caspase-3/7 in this study confirmed apoptosis as the major mechanisms of cell death in U118MG treated with [6]-gingerol as evidenced by the increase of percentages of dead cells with a depolarized mitochondrial membrane. The result maintained was confirmed after 48-hour incubation. Moreover, after 24 and 48-h, we also observed a significant decrease in the percentage of viable cells observed with and increased [6]-gingerol concentration. Research conducted by Lee et al. provided that gingerol positively influences apoptosis GBM cell lines U87, U343, and T98G through TRAIL (TNF-related apoptosis-inducing ligand) [30]. The potential of [6]-gingerol and its synergistic treatment with therapeutic agents has been evaluated for cervical adenocarcinoma, leading to the inhibition growth of HeLa cells, induced cell cycle arrest in the G0/G1 phase, and induced apoptosis [31]. However, in the study on the adenocarcinoma A549 cell line, [6]-gingerol had no effect on cell proliferation [32]; similar results were obtained in our research. Studies carried out on MCF-7 and MDA-MB-231 cell lines after incubation with ginger extract and on prostate cancer cell line PC3R after stimulation with [6]-gingerol with shogalou at 100 μM/mL concentration clearly indicate inhibited cell proliferation [33,34]. The antiproliferative and proapoptotic properties of gingerol on retinal tumor cells were confirmed by Meng et al.; in his study on RB355 cell line, it was found that this antineoplastic effect is due to the ability to induce apoptosis, cell cycle inhibition, and Pi3K/Akt signal regulation [35]. The studies conducted by Lee at al. confirmed anticancer and proapoptotic properties of gingerol; the results indicate that [6]-gingerol has the ability to inhibit cyclin D1, which is protooncogene found in many cancers. This compound induced the expression of NAG-1 proapoptotic cytokine in the colon carcinoma cell line [14]. This also confirms the conclusions of our own research on pro-apoptotic properties of [6]-gingerol. Referring to anticancer effects of silymarin, it was noticed that silymarin inhibited oral tumor growth and proliferation of tumor cells [15,36].

Ranjbar et al. showed that treatment of Ramos cancer cells with 100 μg/mL of sylimarin markedly increased the activity of caspase-3 [37]. Montgomery et al. showed that silymarin at the highest dose of 100 μM/mL has antiproliferative activity in colorectal cancer cell lines: DLD-1, LoVo, and HCT116 [38]. Furthermore, the experiment carried out on the ovarian cell line indicates the inhibitory properties of silymarin: significantly limits the growth and rate of proliferation, inhibits the cell cycle in G1/S phase, and the doses of 50 μg/mL and 100 μg/mL activate the apoptotic pathway and induce cytochrome C release, as well as the reduction of the mitochondrial membrane potential [39]. It was found that 20 μM and 50 μM concentrations almost completely blocked tumor cell invasion, inhibiting cell proliferation and migration of glioblastoma cells. On the other hand, silymarin stimulates the process of apoptosis by activating caspases [40]. It is also a confirmation of the results of our own study, where it was observed that self-acting silymarin induces the death of glioblastoma cells.

## 5. Conclusions

We demonstrated the capacity of lycopene, [6]-gingerol, and silymarin to induce apoptosis and reduction of mitochondrial membrane potential in glioblastoma cells after 48 hours. Moreover, the results obtained from another experiment (data not shown) about stimulatory effects on apoptosis on kidney and lung cancer cell lines confirmed our hypothesis that these compounds may alter cell-cycle regulatory proteins depending on the dose of substances. Taken together, these data indicated that the potential anticancer and proapoptotic effect of lycopene, [6]-gingerol, and silymarin is dose-dependent.

## Supplementary Materials

The following are available online at https://www.mdpi.com/2072-6643/12/1/96/s1, Figure S1: Detection of apoptotic DNA ladder in U118-MG cell line afer 48-hours incubation. Lane M: standard molecular size marker (1 Kb). Lane 1: cells treated with 50 μM silymarin, lane 2: cells treated with 100 μM silymarin, lane 3: cells treated with 200 μM silymarin, lane 4: cells treated with 5 μM lycopene, lane 5: cells treated with 10 μM lycopene, lane 6: cells treated with 50 μM lycopene, lane 7: cells treated with 50 μM [6]-gingerol, lane 8: cells treated with 100 μM [6]gingerol, lane 9: cells treated with 500 μM [6]-gingerol, lane 10: control with 0.5% DMSO.
