# Supplementary material for: The Influence of Lycopene, [6]-Gingerol, and Silymarin on the Apoptosis on U-118MG Glioblastoma Cells In Vitro Model
Source: Nutrients. 2019 Dec 30;12(1):96. doi: 10.3390/nu12010096 (PMC7019537; doi:10.3390/nu12010096)
Supplement: Supplementary file 1 [file nutrients-12-00096-s001.pdf]

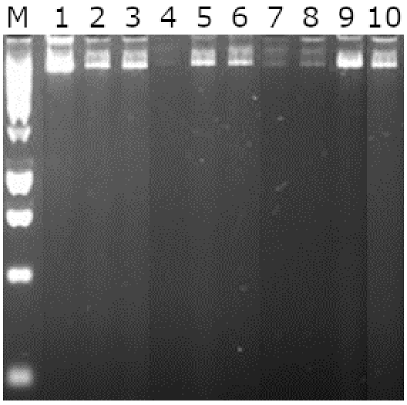

Fig. 1 Detection of apoptotic DNA ladder in U118-MG cell line afer 48-hours incubation. Lane M: standard molecular size marker (1 Kb). Lane 1: cells treated with 50 μM silymarin, lane 2: cells treated with 100 μM silymarin, lane 3: cells treated with 200 μM silymarin, lane 4: cells treated with 5 μM lycopene, lane 5: cells treated with 10 μM lycopene, lane 6: cells treated with 50 μM lycopene, lane 7: cells treated with 50 μM [6]-gingerol, lane 8: cells treated with 100 μM [6]gingerol, lane 9: cells treated with 500 μM [6]-gingerol, lane 10: control with 0.5% DMSO
